# Does the evidence about health risks associated with nitrate ingestion warrant an increase of the nitrate standard for drinking water?

**DOI:** 10.1186/1476-069X-5-26

**Published:** 2006-09-21

**Authors:** Hans JM van Grinsven, Mary H Ward, Nigel Benjamin, Theo M de Kok

**Affiliations:** 1 Dept. of Agriculture and Rural Areas, Netherlands Environmental Assessment Agency, The Netherlands; 2 Division of Cancer Epidemiology and Genetics, National Cancer Institute, NIH, DHHS, Bethesda, MD, US; 3 Derriford Hospital, Derriford, Plymouth, PL68DH, UK; 4 Dept. Health Risk Analysis and Toxicology, University of Maastricht, The Netherlands

## Abstract

Several authors have suggested that it is safe to raise the health standard for nitrate in drinking water, and save money on measures associated with nitrate pollution of drinking water resources. The major argument has been that the epidemiologic evidence for acute and chronic health effects related to drinking water nitrate at concentrations near the health standard is inconclusive. With respect to the chronic effects, the argument was motivated by the absence of evidence for adverse health effects related to ingestion of nitrate from dietary sources. An interdisciplinary discussion of these arguments led to three important observations. First, there have been only a few well-designed epidemiologic studies that evaluated ingestion of nitrate in drinking water and risk of specific cancers or adverse reproductive outcomes among potentially susceptible subgroups likely to have elevated endogenous nitrosation. Positive associations have been observed for some but not all health outcomes evaluated. Second, the epidemiologic studies of cancer do not support an association between ingestion of dietary nitrate (vegetables) and an increased risk of cancer, because intake of dietary nitrate is associated with intake of antioxidants and other beneficial phytochemicals. Third, 2–3 % of the population in Western Europe and the US could be exposed to nitrate levels in drinking water exceeding the WHO standard of 50 mg/l nitrate, particularly those living in rural areas. The health losses due to this exposure cannot be estimated. Therefore, we conclude that it is not possible to weigh the costs and benefits from changing the nitrate standard for drinking water and groundwater resources by considering the potential consequences for human health and by considering the potential savings due to reduced costs for nitrate removal and prevention of nitrate pollution.

## Background

In 2004, the World Health Organization reconfirmed the nitrate standard of 50 mg/l for drinking water which was set to protect against methemoglobinemia. However, some authors [1,2] have questioned the importance of nitrate in drinking water as a risk factor for methemoglobinemia and have suggested that the current standard might be safely raised to 15–20 mg/L nitrate-N (approximately 65–90 mg/l nitrate) with no increase in cases. Other authors [3] reviewed the epidemiologic studies of nitrate and cancer and considered the evidence inconclusive and stated that "nitrate limits could safely be increased to 100 mg/l". Additionally these authors suggested that concern about nitrate in drinking water was another example of what Lomborg [4] defines as "alarms about non-existing threats absorbing financial resources that may be needed to tackle real ones." In contrast, the conclusions of scientists who convened a symposium on drinking water nitrate and health at the International Society for Environmental Epidemiology in 2004 [5] were that "the role of nitrate as a risk factor for cancer and adverse reproductive outcomes must be more thoroughly explored before changes to nitrate water quality standards are considered".

In a subsequent symposium on "The nitrogen cycle and human health", in 2005 the health issues were discussed against the broader context of ecology, food supplies, and energy security. However, the debate returned to the question whether nitrate is really a health threat and whether the costs of measures to deal with nitrate pollution are justified. This has been an ongoing debate for at least three decades in the US and Europe. Although science has made progress, consensus about the health risks associated with nitrate intake, and the need for measures to reduce drinking water nitrate concentrations are far from being resolved. The primary reason for this may be the lack of good interdisciplinary discussions among toxicologists, epidemiologists, environmental scientists, agronomists, clinicians, and policy makers, each of whom plays a different role in the assessment of health risks, and cost-benefits associated with nitrate exposure.

## Discussion

### Chronic effects of drinking water nitrate and dietary nitrate

There is consensus about the likely strong carcinogenic effect of N-nitroso compounds (NOC) in humans based on animal evidence of carcinogenicity in every species tested [6,7]. N-nitroso compounds have been demonstrated to be formed in humans after nitrate ingestion. However, three primary reasons for skepticism for a role of drinking water nitrate in increasing the risk of cancer and other chronic health outcomes are:

1. When nitrate levels in drinking water are below the current regulatory standard, the large majority of individual's nitrate intake is from vegetables rather than water [8]. Therefore, it is likely to be difficult to detect an effect of water nitrate variation because of the widely varying vegetable nitrate intake. The effect of this "noise" in interpreting epidemiological studies has not been taken into account.

2. The half-life of nitrate in the body is over 8 hours, which means that after a meal containing spinach, lettuce or another source of nitrate, the levels in the blood will be elevated for about 40 hours [9]. Also, because of enterosalivary circulation of nitrate, the stomach is subjected to high concentrations of nitrite and nitrate long after the food with its antioxidant protection has disappeared.

3. If nitrate in vegetables is harmless then nitrate in water can only be harmful in those people who eat very little vegetables, so that they have little antioxidant defense. If this is the case, then those individuals whom epidemiological studies have identified who have a high water nitrate intake and have developed cancer because of it will also have a low vegetable intake. It would seem that having a low vegetable intake per se would lead to an increase in cancer susceptibility.

In response to these three reasons for skepticism the following points should be considered:

1. To date, many epidemiologic studies of drinking water nitrate have not considered dietary sources of nitrate [10,5]. Epidemiologic studies that have evaluated dietary sources of nitrate have not provided evidence for a positive association with cancer [10]. Although many epidemiologic studies of drinking water nitrate have not estimated dietary intakes, it is unlikely that dietary nitrate was a confounding factor because in order to be a confounder, the factor has to be associated with both the disease and the exposure (levels of drinking water nitrate). Dietary nitrate intake is not likely to meet these criteria under most situations.

Furthermore, vegetable sources of dietary nitrate are also sources of antioxidant intake, which inhibit the endogenous formation of NOC (nitrosation) [11]. Therefore, to evaluate the risk related to drinking water nitrate exposure it is important to also consider intakes of inhibitors of nitrosation such as antioxidants as well as the ingestion of precursors of NOC formation. The main dietary sources of nitrosatable precursors are meat and fish.

2. It is correct that some antioxidants are excreted or metabolized relatively quickly; however, even if the original dietary antioxidant has been eliminated, the antioxidant effect can still remain as a result of interaction with other cellular antioxidant defense systems. For instance, dietary thiols can increase glutathione levels and vitamins can lower levels of oxidized lipids. Recent human dietary intervention studies with flavonoid-rich foods have shown that the cellular antioxidant potential is still increasing after 3–4 weeks of intervention, indicating that (just like nitrate levels) the antioxidant potential and therefore, the nitrosation inhibiting capacity of specific phytochemicals, are building up over time [12].

3. Nitrate is potentially harmful regardless of the source. Fish contains high levels of nitrosatable precursors, and it has been demonstrated that fish consumption in combination with a nitrate source such as high nitrate vegetables results in increased formation of N-nitroso compounds [13]. Adverse health effects related to endogenously formed NOC are likely to result from the complex interaction of the amount of nitrate ingested, the concomitant ingestion of NOC precursors and inhibitors of nitrosation, and other cofactors in the nitrosation process such as heme iron from red meat [14] and medical conditions that may increase endogenous nitrosation (e.g. chronic inflammatory diseases [15]).

To date, there have been few epidemiologic studies of any cancer site that have evaluated drinking water nitrate exposure among potentially susceptible subgroups with higher nitrosation. An association between specific cancers and ingestion of drinking water nitrate, particularly among individuals with low fruit and vegetable consumption is plausible and has been described previously for colon cancer [16]. Other epidemiologic studies have observed increased cancer risk among those using public water supplies with elevated nitrate levels for several decades, although water nitrate concentrations were below the regulatory standard. Elevated risks associated with higher public supply nitrate levels were observed for non-Hodgkin's lymphoma [17], urinary bladder, and ovarian cancer [18]. However, other studies with similar exposure levels found no association for non-Hodgkin's lymphoma [19] and bladder cancer [20]. The study by Weyer et al [18] also reported inverse relationships between drinking water nitrate and uterine and rectal cancer.

Several studies suggest an association between drinking water nitrate ingestion and congenital malformation particularly neural tube defects (reviewed in [21]; [5]) although the evidence for a causal role for nitrate in specific adverse reproductive outcomes is not conclusive. Brender et al [22] found that ingestion of nitrate in food or water significantly modified the risk associated with use of nitrosatable drugs during the periconceptual period. Higher nitrate ingestion significantly increased the risk of neural tube defects if the women used nitrosatable drugs, which include many common prescription and nonprescription medications.

Most of the epidemiologic studies to date have limitations and more studies that address the complexities of endogenous nitrosation in humans are needed. Two additional directions that may be fruitful are to conduct a meta-analysis of the epidemiologic studies and to conduct additional kinetic studies of endogenous nitrosation and NOC-metabolism. Meta-analyses are valuable tools for generating conclusions about specific chronic health effects (e.g., stomach cancer, colon cancer, bladder cancer, specific reproductive outcomes). However, the number of suitable studies for any particular health effect is presently too small to conduct meta-analyses. Furthermore, high drinking water nitrate in combination with other risk factors may stimulate endogenous nitrosation and exposure to NOC. For instance, patients with bilharzias have an increased bladder cancer risk associated with increased urinary levels of nitrite and volatile nitrosamines, most likely generated by the reaction of inflammation derived NO with amines present in the urine. Inflammatory bowel disease is also related to both increased nitrosation and cancer risk, whereas increased levels of faecal NOC have been found in patients with inflammatory bowel disease and in mice with chemically induced colitis [5,15].

Future kinetic studies would be useful to establish an improved quantitative relationship between nitrate intake and endogenous exposure levels of NOC as well as to develop validated biomarkers of such exposures to be applied in future epidemiological studies taking susceptible subpopulations into account.

### Exposure to nitrate in drinking water and associated health loss

One conclusion of this interdisciplinary discussion was an agreement that there are both experimental and epidemiologic studies that indicate possible chronic health effects associated with consumption of elevated levels of drinking water nitrate, although there is no consistency across all studies. Therefore, the uncertainties associated with risk estimates are considerable, and hamper the design of cost-effective specific preventive measures for sensitive subpopulations or regions. Moreover, the enhanced risk of NOC-induced toxicity as a result of high drinking water nitrate in combination with other individual risk factors, such as inflammatory diseases, emphasizes the importance of changing the limit values only when such risks have been carefully evaluated. At this moment this is not the case. Likewise, uncertainties do not allow an estimate of the health losses related to methemoglobinemia due to drinking water nitrate [23]. Therefore, the costs of lowering drinking water nitrate levels cannot be compared with potential health benefits. In situations when the health effects are more clearly related to an exposure, such as for the relationship between mortality and exposure to airborne particulate matter, cost-benefit studies assume a value around 50 thousand euros for the loss of a healthy life year in Western Europe and North America. This led to estimates for the European Union of total health loss associated with airborne particulate matter of 90–190 billion euro per year. This outcome greatly accelerated policies to set standards and implement preventive measures.

**Figure 1:**
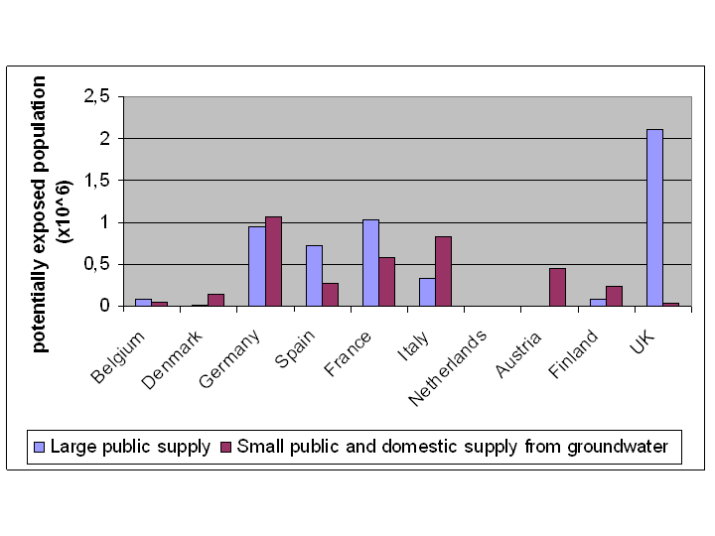
**Exposure to > 50 mg/l nitrate in EU drinking water**. Number of people in 10 EU member states potentially exposed to drinking water exceeding the WHO standard for nitrate and nitrite, considering large public supply companies, and private or small public groundwater wells.

Although it is not yet possible to estimate health loss due to nitrate, it is possible to make estimates of potential exposure. Based on data reported to the European Commission [24] about the implementation of the Drinking Water Directive (Table 1) and data on the present nitrate levels in groundwater at drinking water extraction depths [25], the population in ten west European countries potentially exposed to drinking water exceeding the 50 mg/l nitrate standard, or the 3 mg/l nitrite standard, was estimated at over 9 million (2,7%) (Figure 1).

**Table 1:** Some characteristics about drinking water supply and nitrogen sources in the EU and US.

	Population		Drinking water supply			Waste water	N-surplus agriculture
	
	million	%rural	%ground water	%surface water	%large^a ^facilities	%untreated	kg/ha
Austria	7.8	46	95	5	60	14	36
Belgium	10.2	4	53	33	90	62	145
Denmark	5.3	39	99	1	74	11	111
Finland	5.3	63	34	36	36	19	56
France	59.7	17	64	36	73	23	41
Germany	82.7	14	72	16	82	7	92
Greece	10.7	40	50	50	69	44	48
Ireland	3.6	71	25	75	75	27	63
Italy	57.6	10	85	15	83	31	40
Netherlands	15.9	2	66	34	100	2	256
Spain	28.4	16	35	65	73	45	35
UK	58.1	4	27	58	98	5	37
US^b^	293.7	20	33	67	84	no data	^c^43

a) serving more than 5000 consumers,

b) US data from [34], EU data from [22] and EUROSTAT

c) Inferred from [35]

This number is comparable to an estimate for the US of nearly 6 million (2%) [26]. The most recent data in the US indicate that about 22% of domestic wells in agricultural areas of the US and 3% of public supply wells in major aquifers (typical sources for public water supplies) exceed the standard (U.S. Geological Survey, unpublished data). The estimates for the US and Europe are far lower than those for some developing countries such as India, where it is estimated that 13 million people (17%) in the state of Andra Pradesh consume drinking water above the regulatory standard [27]. These exposure estimates give some indication of present potential health loss and potential benefits of preventive measures. Severe methemoglobinemia has been documented in India [28] related to elevated nitrate in drinking water. However, other health effects related to the serious water sanitation problems in India are likely to be more important for estimating health losses [27]. Reported incidence of methemoglobinemia in Europe is low [29].

In the US and Europe, actual exposure to nitrate in drinking water may be much lower than estimates based on levels in water resources because of the increasing consumption of bottled water. The use of bottled water is largely a market phenomenon, but in drinking water supply areas with high nitrate concentrations it is recommended to use bottled water to prepare infant formula and baby food [29]. Annual costs of unnecessary consumption of bottled water could amount to several hundreds of euros or dollars per household. These costs, and also costs for installing new domestic and public drinking water wells, can be far higher than costs for preventing contamination or for removal of nitrate from drinking water. Nitrate in groundwater may also mobilize toxic trace metals (e.g. nickel) by oxidation of metal containing sulfides (e.g. pyrite), possibly resulting in indirect adverse health effects as a result of elevated nitrate in drinking water [30].

In addition to potential adverse health effects, an important reason to prevent nitrate contamination of water resources is to avoid the adverse ecological effects of nitrate and excess nitrogen in surface water ecosystems. In fresh water systems, the toxic effect of nitrate occurs by a similar mechanism to that causing methemoglobinemia in humans. Toxicity increases with decreasing body size and environmental adaptation potential [31]. The recommended level to protect invertebrates is 10 mg/l nitrate (2 mg/l NO3-N), which is 5 times lower than the drinking water standard. Levels to ensure a diversity of submerged plant life in freshwater lakes also may be as low as 1–2 mg/l [32]. Nitrogen is a key element in determining biodiversity when phosphate levels are sufficiently low to prevent algae or duckweed blooms. Therefore, it is probable that the detrimental effects of nitrogen inputs from agriculture and other human sources on fresh water ecosystems will force stricter limits on nitrate levels in water resources than limits based on concerns related to human consumption. Exposure to toxins released during algae blooms resulting from eutrophication is another potential health hazard for humans related to elevated nutrient levels in surface waters [33].

## Conclusion

In summary, additional well-designed health studies are needed in order to estimate the potential health losses related to consumption of drinking water nitrate at the current health standard. Furthermore, additional exposure data and an understanding of exposure response relationships are needed in order to conduct a full health risk assessment and determine the potential economic gain associated with increasing the nitrate standard for drinking water and groundwater resources.

## Competing interests

The author(s) declare that they have no competing interests.

## Authors' contributions

HJMG is an agro-environmental scientist who participated at the symposium on the nitrogen cycle and human health, in November 2005 in Salt Lake City. HJMG initiated the paper and was responsible for the section "Exposure to nitrate in drinking water and associated health loss" and integrated the contributions. NB is a clinician and admitted skeptic against health risks of nitrate in drinking water. He also was present in Salt Lake City and he formulated the major objections to the epidemiologic evidence. MHW and TMK are epidemiologists and toxicologists, respectively, and co-authors of the paper summarizing the symposium on drinking water nitrate and health, in August 2004 in New York City. MHW and TMK were responsible for response to the questions raised by NB in the section "Chronic effects of drinking water nitrate and dietary nitrate".
